# AAV8 Ins1-Cre can produce efficient β-cell recombination but requires consideration of off-target effects

**DOI:** 10.1038/s41598-020-67136-w

**Published:** 2020-06-29

**Authors:** Adam Ramzy, Eva Tudurí, Maria M. Glavas, Robert K. Baker, Majid Mojibian, Jessica K. Fox, Shannon M. O’Dwyer, Derek Dai, Xiaoke Hu, Heather C. Denroche, Nazde Edeer, Sarah L. Gray, Cameron B. Verchere, James D. Johnson, Timothy J. Kieffer

**Affiliations:** 1 Department of Cellular and Physiological Sciences, Life Sciences Institute, University of British Columbia, Vancouver, British Columbia Canada; 2 Centro de Investigación Biomédica en Red de Diabetes y Enfermedades Metabólicas Asociadas (CIBERDEM), Madrid, Spain; 3 Instituto de Investigación, Desarrollo e innovación en Biotecnología Sanitaria de Elche (IDiBE), Elche, Spain; 4 Department of Surgery, University of British Columbia, Vancouver, British Columbia Canada; 5 Northern Medical Program, University of Northern British Columbia, Prince George, British Columbia Canada; 6 Department of Pathology and Laboratory Medicine, BC Children’s Hospital Research Institute, Vancouver, British Columbia Canada

**Keywords:** Genetic engineering, Gene delivery

## Abstract

*In vivo* genetic manipulation is used to study the impact of gene deletion or re-expression on β-cell function and organism physiology. Cre-LoxP is a system wherein LoxP sites flanking a gene are recognized by Cre recombinase. Cre transgenic mice are the most prevalent technology used to deliver Cre but many models have caveats of off-target recombination, impaired β-cell function, and high cost of animal production. Inducible estrogen receptor conjugated Cre models face leaky recombination and confounding effects of tamoxifen. As an alternative, we characterize an adeno associated virus (AAV) with a rat insulin 1 promoter driving Cre recombinase (AAV8 Ins1-Cre) that is economical and rapid to implement, and has limited caveats. Intraperitoneal AAV8 Ins1-Cre produced efficient β-cell recombination, alongside some hepatic, exocrine pancreas, α-cell, δ-cell, and hypothalamic recombination. Delivery of lower doses via the pancreatic duct retained good rates of β-cell recombination and limited rates of off-target recombination. Unlike inducible Cre in transgenic mice, AAV8 Ins1-Cre required no tamoxifen and premature recombination was avoided. We demonstrate the utility of this technology by inducing hyperglycemia in inducible insulin knockout mice (*Ins1*^−/−^;*Ins2*^f/f^). AAV-mediated expression of Cre in β-cells provides an effective alternative to transgenic approaches for inducible knockout studies.

## Introduction

Diabetes is a chronic condition affecting over 400 million worldwide and is characterized by a relative or absolute insulin insufficiency associated with a loss of insulin secreting pancreatic β-cells. As such, the development and function of β-cells is a major focus of diabetes research. Specific *in vivo* genetic modification is a useful way to assess the impact of genes on relevant physiological processes and one of the most useful genetic tools to study the role of specific genes is the Cre-LoxP system. Cre recombinase is an enzyme that recognizes LoxP sites in the genome and based on orientation and location, can excise, flip, or translocate targets. By using a tissue specific promoter for Cre recombinase, LoxP flanked sites can be deleted in a tissue-specific manner^1^. Additionally, recombination can be temporally controlled by conjugating Cre to a modified estrogen receptor (ER). By fusing Cre to an ER, Cre is retained in the cytoplasm and is thus unable to bind DNA until the tamoxifen metabolic products endoxifen or 4-hydroxytamoxifen bind the ER and translocate Cre to the nucleus. Many Cre driver mouse lines have been generated, including dozens that are specific for the pancreas or certain pancreatic cell lineages^2^. These tools have been used extensively with great success, but this approach faces important caveats.

One of the earliest pancreatic Cre driver mice developed uses 668 bp of the rat insulin 2 (*Ins2*) promoter to drive Cre, often called the “RIP-Cre” mouse^3^. This mouse model has been used in many studies, but findings have been challenged because of a lack of appropriate controls in many studies. There may be developmental defects from lifelong Cre expression and there are potential impairments in insulin secretion and glucose tolerance in Ins2-Cre transgenic mice^4^. Moreover, β-cells of “RIP-CreER” (Ins2-cre/Esr1) mice have increased rates of apoptosis in response to glucagon-like peptide 1 stimulation^5^. Additionally, an important caveat faced by almost all Cre driver lines is imperfect tissue specificity of recombination. Cre and CreER driver lines using the *Ins2* promoter have abundant recombination throughout the brain^6,7^. Alternative lines make use of a pancreatic and duodenal homeobox 1 gene (*Pdx1*) promoter to control Cre expression, but it has been reported to induce hypothalamic^7^, α-cell, and δ-cell recombination^8^. To address this issue, a more specific *Ins1* promoter has been used and may limit these off-target confounding effects^9,10^. Further, inducible ER conjugated Cre models like the Ins2-cre/Esr1 mouse can have tamoxifen-independent recombination^11^ but this can be limited by use of the mutated ER in Cre-ER^T2^^12^. However, it is notable that delivery of tamoxifen itself (to induce recombination) can alter glucose homeostasis and impair β-cell proliferation^13^. Additionally, inclusion of a growth hormone (GH) minigene in many Cre transgenic driver lines (including Ins2-Cre, Pdx1-Cre^Late^, Ins1-Cre and others) is also a source of β-cell dysfunction via local activation of prolactin receptors^14^ that can induce β-cell proliferation^15^. Furthermore, recent studies suggest that DNA hypermethylation of the Ins1^Cre^ and Ins1^CreER^ knock-in alleles in some colonies can lead to very poor rates of recombination and it may be necessary to validate Cre efficiency regularly^16^. Finding a way to avoid caveats of developmental deletion of LoxP flanked genes, GH minigene-induced β-cell dysfunction, unintended recombination before tamoxifen administration, and tamoxifen toxicity would be useful. It is also worth noting the time and costs of crossing Cre driver mice with LoxP containing mice to generate mice with suitable genotypes. Finding ways to minimize these costs and delays could make complex genetic studies more accessible to laboratories that face time, labour, or financial challenges.

AAV is a well-known and simple vector that could be an alternative approach to deliver Cre to pancreatic β-cells in a specific and cost-effective way. AAVs have been used in many fields to deliver genes of interest to most tissues of the body including in hundreds of clinical trials^17^. AAV is considered non-pathogenic^18^ and infection does not cause any easily detected or common symptoms^19^. Gene expression is initiated less than 2 weeks after infection and there have been reports of residual expression up to four years after therapy in a patient^20^. Each AAV serotype has a distinct tropism for a variety of tissues^21^ and the eighth serotype (AAV8) has the highest affinity for the pancreas when delivered by intraperitoneal (IP) or intravenous injection^22^. AAV delivered via the pancreatic duct at ~1/10^th^ the dose of IP delivery can infect β-cells at comparable efficiency^23^.

In the current study we characterize an AAV8 Ins1-Cre for delivery of Cre recombinase to pancreatic β-cells. We delivered variable doses of AAV8 Ins1-Cre and assessed β-cell function and maturity and demonstrate the utility of this AAV by inducing diabetes in *Ins1*^−/−^*;Ins2*^*f/f*^ mice. Though off-target recombination events likely occurred throughout the liver and exocrine pancreas, we posit that this would have little to no impact because these cells do not normally produce insulin. We avoided months of crossing and genotyping, tamoxifen administration, and lifelong Cre expression. In addition, animals served as their own controls by comparing pre and post AAV injection, and there was no recombination prior to AAV8 Ins1-Cre injection.

## Results

### Intraperitoneal administration of AAV8 Ins1-Cre up to a dose of 1 × 10^12^ VGP (viral genome particles) does not significantly alter glucose metabolism

**Figure 1:**
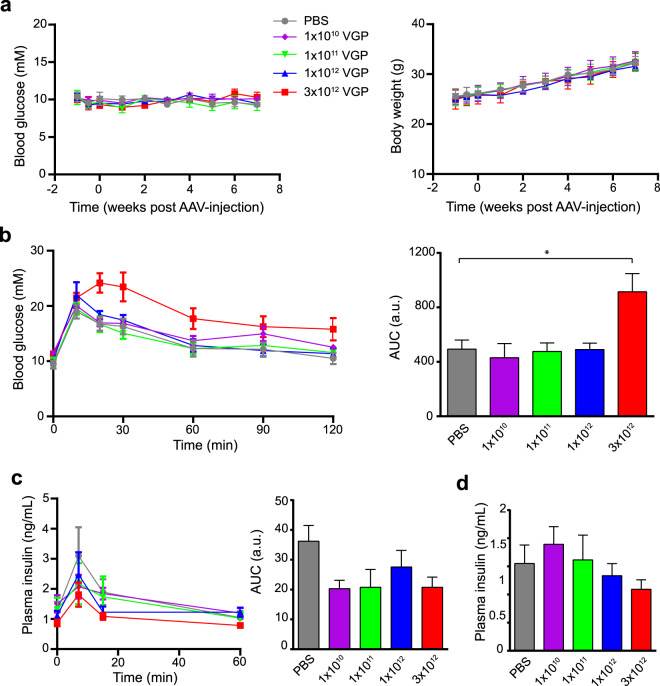
Intraperitoneal administration of AAV8 Ins1-Cre up to a single dose of 1 × 10^12^ VGP does not significantly alter glucose metabolism. (**a**) 4-hour fasting blood glucose and body weight of adult mTmG reporter mice injected with either vehicle (PBS) or different doses of AAV8 Ins1-Cre. (**b**) Oral glucose tolerance test (6 hour fast, 2 g glucose/kg body weight) 7 weeks post-virus injection. Area under the curve (AUC) relative to baseline is presented to the right in arbitrary units (a.u.). (**c**) Plasma insulin levels during oral glucose tolerance test and (**d**) after 6 hours fasting 8 weeks post-virus injection. Data are expressed as mean ± SEM and were analyzed using one- or two-way repeated measures ANOVA. n = 4–5 mice per group. (*p < 0.05).

To determine whether AAV8 Ins1-Cre (410 bp of the rat *Ins1* promoter, intron, and the full Ins1 5′UTR sequence preceding the Cre open reading frame) leads to Cre expression and subsequently Cre recombination in β-cells, we administered AAV8 Ins1-Cre at doses of 1 × 10^10^, 1 × 10^11^, 1 × 10^12^, and 3 × 10^12^ VGP by a single IP injection to adult reporter mTmG mice. This double fluorescent reporter model contains a chicken β-actin core promoter with a CMV enhancer driving a loxP-flanked coding sequence of membrane-targeted tandem dimer Tomato (mT), resulting in tdTomato expression. Upon Cre recombination, the mT sequence is excised allowing expression of membrane-targeted EGFP (mG)^24^. A group of PBS-injected mTmG mice were used as controls. As several reports have already shown that transgenic Ins2-Cre mice may develop glucose intolerance^4^, we studied glucose metabolism over 8 weeks (−1 to 7 weeks relative to AAV administration). There were no significant changes to four hour fasting blood glucose and body weight between the AAV8 Ins1-Cre injected mice and controls (Fig. 1a). Seven weeks after administration, we performed an oral glucose tolerance test (OGTT, 2 g/kg body weight of a 30% dextrose solution) and found that delivery of AAV8 Ins1-Cre up to a dose of 1 × 10^12^ VGP did not alter glucose excursions; however, 3 × 10^12^ VGP caused glucose intolerance (Fig. 1b). Eight weeks after AAV injections, we assessed insulin secretion during a glucose challenge and there were no significant differences among groups (Fig. 1c). Basal plasma insulin levels were similar between the AAV8 Ins1-Cre and PBS injected groups (Fig. 1d). In addition, we measured serum levels of alanine transferase (ALT) and aspartate aminotransferase (AST) pre- and post-virus administration (Supplemental Fig. 1) and did not detect elevated serum transaminases in AAV8 Ins1-Cre mice compared to controls.

### Intraperitoneal administration of AAV8 Ins1-Cre (1 × 10^12^ VGP) does not significantly alter calcium nor insulin secretion dynamics in isolated islets

**Figure 2:**
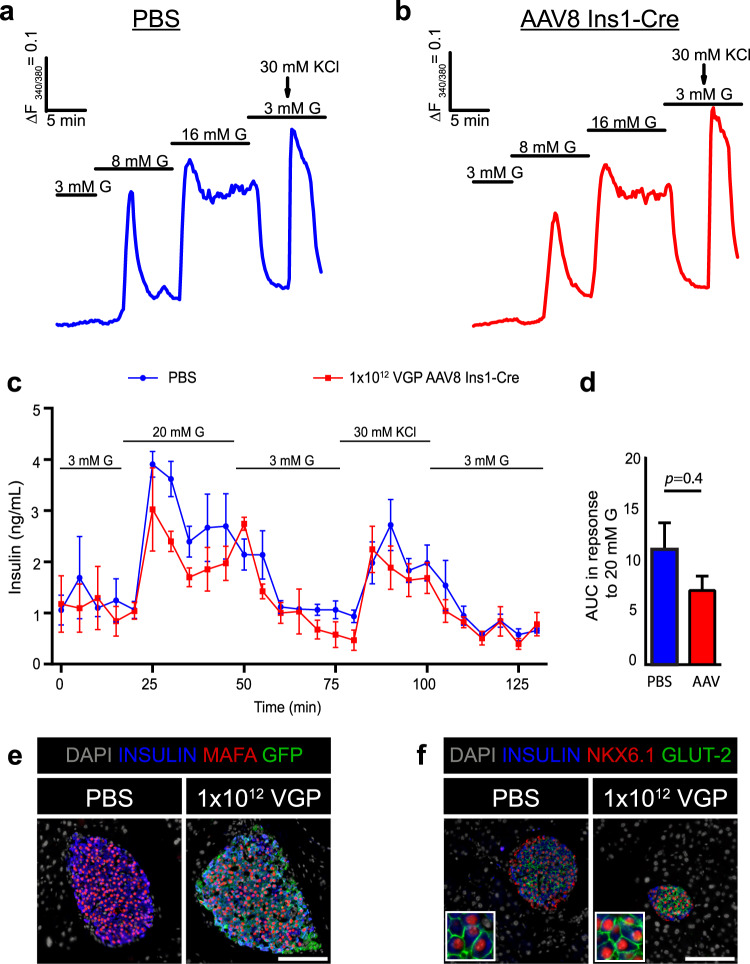
Intraperitoneal administration of AAV8 Ins1-Cre (1 × 10^12^ VGP) does not significantly alter β-cell maturity or function. Adult C57BL/6 J mice were given either PBS or 1 × 10^12^ VGP of AAV8 Ins1-Cre by single intraperitoneal injection, and islets were isolated three to four weeks post-injection. Representative [Ca^2+^]_i_ recordings of an islet from a PBS treated mouse (**a**) and an AAV8 Ins1-Cre injected mouse (**b**), in response to different glucose (G) concentrations and potassium chloride (KCl). Graphs are representative of 31–47 islets from 3 mice per group. (**c**) Insulin secretion in response to G and KCl in islets from PBS and AAV-injected mice, and (**d**) area under the curve (AUC) in response to high glucose (20 mM G) from islets perifused in (**c**). Batches of 80 islets from 3 mice per group were employed. Mann-Whitney test was performed. Pancreas from adult mTmG mice injected with PBS or AAV8 Ins1-Cre was immunostained for mature β-cell factors MAFA (**e**), NKX6.1, and GLUT-2 (**f**). Representative images of n = 3 shown. Scale bars are 100 μm.

Cre-expressing β-cells may display altered intracellular Ca^2+^ responses when exposed to different stimuli including glucose and KCl^25^. We performed intracellular Ca^2+^ imaging experiments in islets from AAV8 Ins1-Cre- and PBS-treated wildtype C57BL/6 J mice using Fura 2-AM to determine if Cre expression mediated by AAV8 Ins1-Cre altered intracellular Ca^2+^ signalling. We used wild-type mice because we observed abnormal Ca^2+^ responses in untreated mTmG islets which is at least partially attributable to overlapping emission spectra of Fura 2-AM and the mTmG fluorescent proteins (data not shown). Using a dose of 1 × 10^12^ VGP, we carried out Ca^2+^ experiments three to four weeks post-injection of either PBS or 1 × 10^12^ VGP of AAV8 Ins1-Cre. Islets from AAV8 Ins1-Cre injected mice did not display obviously altered intracellular Ca^2+^ dynamics in response to elevated glucose concentrations (8 and 15 mM) or in response to depolarizing 30 mM KCl (Fig. 2a,b). In addition, we observed no significant differences in insulin secretion when comparing islets isolated from PBS and AAV8 Ins1-Cre injected mice (Fig. 2c,d). Similar to controls, β-cells of AAV8 Ins1-Cre injected mice had bright nuclear MAFA immunoreactivity (Fig. 2e). Additionally, β-cells in AAV8 Ins1-Cre injected mice had nuclear NKX6.1, and membranous GLUT-2 immunoreactivity (Fig. 2f), as expected based on observations of normal glucose responsive insulin secretion dynamics.

### Intraperitoneal AAV8 Ins1-Cre produces robust β-cell recombination alongside off-target hypothalamic and exocrine pancreas recombination

**Figure 3:**
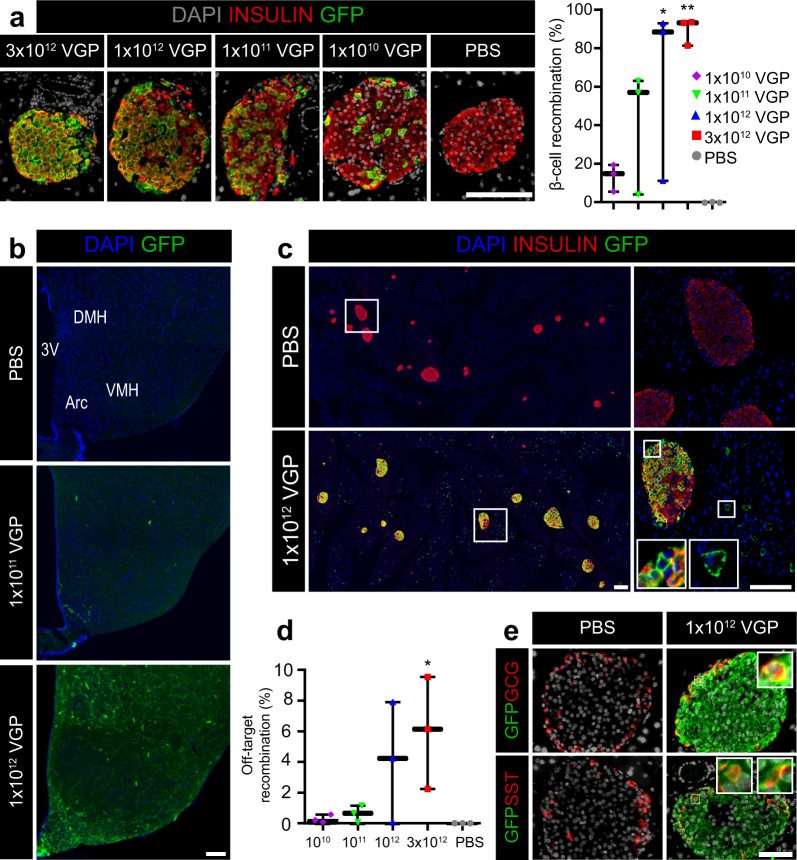
Intraperitoneal AAV8 Ins1-Cre produces dose-dependent β-cell recombination alongside hypothalamic and acinar tissue recombination. Pancreas from mTmG mice administered PBS or varying doses of AAV8 Ins1-Cre was collected 10 weeks post-AAV and immunostained for insulin and GFP (**a**), and recombination rate in β-cells was quantified (n = 3). Groups were compared to PBS control by Kruskal-Wallis test with Dunn’s post hoc test. (**b**) Brains from these mice were immunostained for GFP with nuclear DAPI counterstain. A labeled coronal section through the mid-hypothalamus is shown (3 V: third ventricle, ARC: arcuate nucleus, VMH: ventromedial nucleus of the hypothalamus, and DMH: dorsomedial nucleus of the hypothalamus). Representative image of n = 3. (**c**,**d**) Significant exocrine and insulin negative islet cell recombination in AAV8 Ins1-Cre injected mice. Representative images of n = 3 shown. Quantification of the proportion of insulin negative cell recombination was compared to PBS control by one-way ANOVA with Tukey’s post-hoc test. (**e**) Pancreas was immunostained for GFP and the islet hormones glucagon (GCG) or somatostatin (SST). Representative images of n = 3 shown. Scale bars in all panels are 100 μm. Insets are enlarged 4×. (*p < 0.05, **p < 0.01).

We first demonstrated high recombination efficiency with AAV8 Ins1-Cre by infecting islets from mTmG mice *in vitro*. We observed bright GFP signal throughout the islet for 4 days post-infection (Supplemental Fig. 2). We next immunostained pancreas from AAV8 Ins1-Cre injected mice for insulin and GFP (Fig. 3a). There were high rates of β-cell recombination (81–93%) in 3/3 mice in the 3 × 10^12^ VGP and 2/3 mice in the 1 × 10^12^ VGP dosed mice. There were expected lower rates of β-cell recombination in lower-dosed mice with essentially zero recombination in the PBS-treated control that has never been exposed to any Cre recombinase (Fig. 3a). As there are hypothalamic cells that express *Ins2*^26^ and AAV is known to infect the brain^27^, we immunostained sections of hypothalamus (Fig. 3b). Although there was rare recombination in low (1 × 10^11^) VGP-dosed mice, there was expansive recombination throughout the hypothalamus of 1 × 10^12^ VGP-dosed mice, particularly in neurons of the arcuate and dorsomedial nucleus but not the ventromedial nucleus of the hypothalamus. Additionally, we observed abundant acinar cell recombination and occasional recombination in non-β cells within islets (Fig. 3c,d) that were immunoreactive for glucagon (α-cells) or somatostatin (δ-cells; Fig. 3e). Rates of recombination were dose-dependent with upwards of 10% of insulin-negative cells in pancreatic sections being GFP + . As a potential mechanism of activation of the Ins1 promoter fragment in the AAV8 Ins1-Cre, δ-cells express the insulin promoter binding transcription factor PDX1 (*Pdx1*)^28^ and others have reported detectable expression of *Pdx1* in 5% of mouse acinar cells in the pancreas with 3/182 acinar cells having *Pdx1* levels higher than the mean in β-cells^29^. Notably, GFP expression in this model functions on a binary system: present (Cre has recombined mTmG) or absent (no recombination event and cells continue to make tdTomato). A recombination event from a tiny amount of off-target Cre is sufficient to produce equivalent GFP signal to a cell with robust Cre expression, in contrast to other models (such as transduction with AAV8 Ins1-GFP) in which there can be a wide gradient of GFP expression and weak off-target expression may not be detectable.

### Intraductal (ID) administration of AAV8 Ins1-Cre up to a dose of 1 × 10^11^ VGP does not significantly affect body weight, blood glucose levels or glucose tolerance

**Figure 4:**
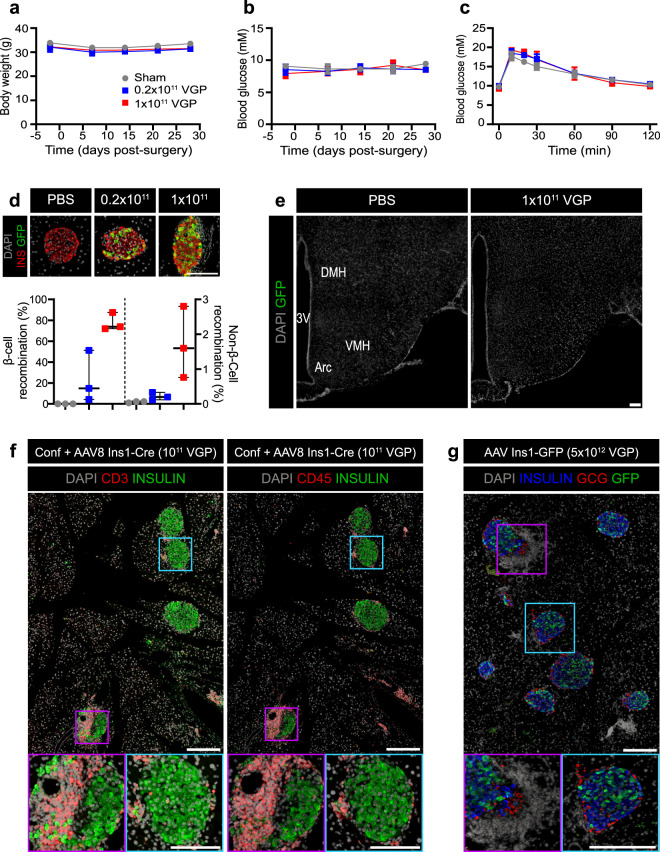
AAV8 Ins1-Cre intraductal administration does not significantly alter glucose tolerance but induction of foreign fluorescent protein expression causes insulitis. Four hour fasting body weight (**a**) and blood glucose (**b**) of adult confetti reporter mice that received AAV8 Ins1-Cre by intraductal delivery. (**c**) Oral glucose tolerance test (2 g glucose/kg body weight) 4 weeks post virus administration. Data are expressed as mean ± SEM and were analyzed using two-way repeated measures ANOVA. n = 7–8 mice in each group (**a**–**c**). Pancreas was collected seven weeks post-AAV and immunostained for insulin (INS) and GFP, and proportion of INS+ or INS- cells that were GFP + were quantified (**d**). Representative images of n = 3 shown and individual animal data points shown. Groups were compared to PBS control by Kruskal-Wallis test with Dunn’s post hoc test. (**e**) Brains were immunostained for GFP and a coronal section through the mid-hypothalamus is shown (3 V: third ventricle, ARC: arcuate nucleus, VMH: ventromedial nucleus of the hypothalamus, and DMH: dorsomedial nucleus of the hypothalamus). Representative image of n = 1 of control or n = 3 of 1 × 10^11^ VGP injected mice shown. There was no detectable GFP in 0.2 × 10^11^ VGP dosed mice (data not shown). (f) Adult confetti mice injected with AAV8 Ins1-Cre had insulitis with CD3 + and CD45 + cells surrounding islets. Representative images of n = 3 shown. (**g**) Adult C57BL/6 J mice were injected with 5 × 10^12^ VGP AAV8 Ins1-GFP IP and developed insulitis by 4 months post-AAV. Representative images of n = 2. Scale bars are 100 μm.

To minimize central recombination and other potential off-target sites observed following IP delivery, we delivered AAV8 Ins1-Cre via the common bile duct (ID). In this study, we used adult confetti reporter mice that express one of four fluorescent proteins upon Cre-mediated recombination events^30^. Body weight and fasting blood glucose measured over a period of 35 days were not significantly different among the sham group and the AAV8 Ins1-Cre injected animals (0.2 × 10^11^ VGP and 1 × 10^11^ VGP groups; Fig. 4a,b). Four weeks post-surgery, there were no significant differences in oral glucose tolerance (Fig. 4c). Pancreas was collected six weeks post-AAV and immunostained for insulin and fluorescent proteins. As the four fluorescent proteins of the confetti mouse have extremely high sequence similarities, we immunostained with a polyclonal GFP antibody or a monoclonal antibody to GFP and a polyclonal dsRed antibody to provide sufficient sensitivity to detect all recombination events (Supplemental Fig. 3). We detected three distinct immunostaining patterns that likely represent all fluorescent proteins with the exception of nuclear GFP, which is reported to be less common or absent in many tissues including pancreas of confetti mice^30^. Using the polyclonal GFP antibody, we quantified the rates of β-cell and non-β-cell recombination in the pancreas and found significant recombination in 1 × 10^11^ VGP injected mice (Fig. 4d). We immunostained brain and found no evidence of recombination in the hypothalamus (Fig. 4e), in contrast to our findings with IP delivery of AAV8 Ins1-Cre (Fig. 3b). Notably, histological experiments identified significant accumulation of DAPI + Ins- cells near islets that resembled insulitis. We immunostained for the T-cell co-receptor CD3 and pan-leukocyte protein CD45 and confirmed heterogeneous insulitis (Fig. 4f). We similarly observed insulitis in another different model that is naïve to fluorescent protein expression into adulthood until IP delivery of a high dose AAV8 Ins1-GFP (Fig. 4g). In AAV8 Ins1-GFP injected mice we noted bright GFP signal in targeted β-cells (Fig. 4g), but no detectable acinar GFP, in contrast to the widespread acinar, α-cell, and δ-cell GFP expression observed following IP administration of AAV8 Ins1-Cre (Fig. 3c). These findings reveal that ID delivery of AAV8 Ins1-Cre limits off-target recombination in the hypothalamus and again highlights the binary outcome in the mTmG or any other lineage trace model employing a Cre transgene.

### Both IP and ID administration of AAV8 Ins1-Cre can cause recombination in the liver

**Figure 5:**
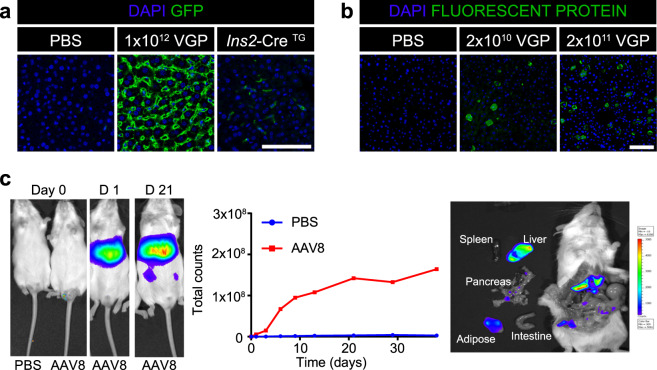
Both IP and ID administration of AAV8 Ins1-Cre can cause recombination in the liver but the extent may be dependent on delivery method and mouse strain. Liver from adult mTmG mice given IP AAV8 Ins1-Cre (**a**) or confetti mice given ID AAV8 Ins1-Cre (**b**) was immunostained for fluorescent proteins to identify hepatic recombination events. Representative images of n = 2–3 shown. Scale bars are 100 μm. (**c**) Adult Rosa26-LSL-luciferase mice were administered 1 × 10^12^ VGP AAV8 Ins1-Cre IP (n = 3) or PBS (n = 1) and luciferase activity was assessed following luciferin injection for 5 weeks including after rapid dissection at study termination.

As AAV8 has a high propensity to infect hepatocytes^22^, we looked for evidence of recombination in the liver. There was robust recombination in hepatocytes in the IP AAV8 Ins1-Cre injected mTmG mice (Fig. 5a). We also observed some less abundant recombination in transgenic Ins2-Cre;mTmG mice and based on nuclear and cell morphology, as well as published single cell RNA seq data sets^29^, these GFP + cells could be endothelial cells of the sinusoids or resident Kupffer cells, rather than hepatocytes. There were low rates of recombination in the liver of mice given AAV8 Ins1-Cre via the pancreatic duct (Fig. 5b). To further explore liver recombination, we delivered 1 × 10^12^ VGP AAV8 Ins1-Cre IP to Rosa26-LSL-luciferase mice. When Cre is present in Rosa26-LSL-luciferase mice, Cre removes the LSL (loxP-transcriptional stop site-LoxP) sequence to allow transcription of luciferase. We performed *in vivo* and *ex vivo* chemiluminescent experiments following luciferin injection (Fig. 5c). There was significant luminescence for five weeks post-AAV and by dissecting out organs quickly after luciferin injection, it was revealed that the majority of activity was coming from the liver and minority from the pancreas and adipose tissue. Though early reports using AAV8 chicken β-actin promoter-GFP^22^ would suggest the pancreas has the highest expression levels, these findings again highlight the binary outcome of a lineage tracing model like this luciferase mouse. Past work reveals that total expression of an AAV8 is likely highest in the pancreas (i.e. measuring GFP from an AAV actin-GFP)^22^ but the current work suggests that the IP infection rate is highest in the liver.

### Deletion of a β-cell specific gene with AAV8 Ins1-Cre

**Figure 6:**
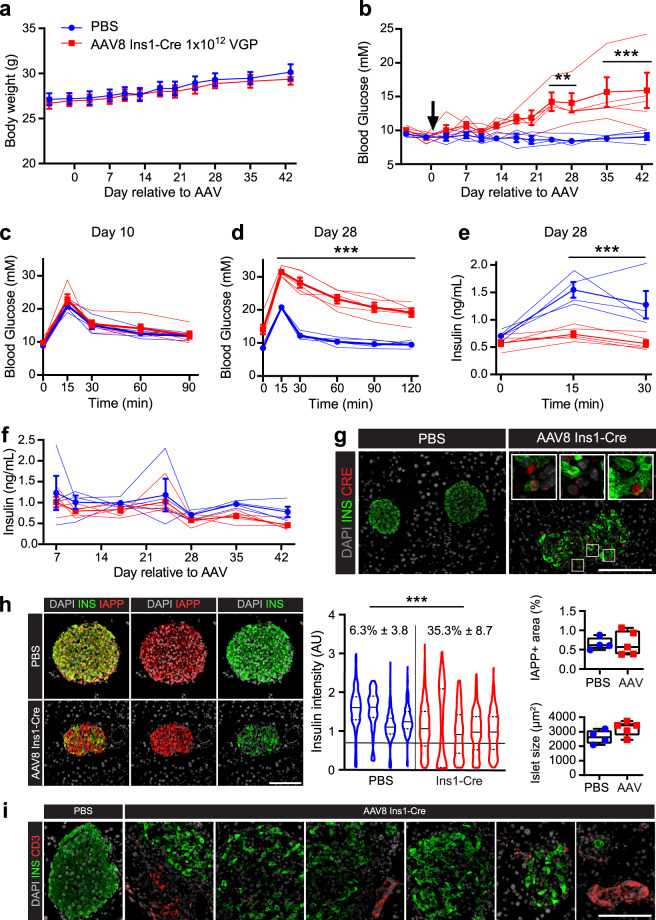
The AAV8 Ins1-Cre can be a useful tool for directing β-cell recombination when off-target effects are deemed minimally important. Four hour fasting body weight (**a**) and blood glucose (**b**) of adult *Ins1*^−/−^*;Ins2*^*f/f*^ mice that received AAV8 Ins1-Cre by IP delivery (indicated by black arrow). IPGTTs were performed on day 10 (**c**) and day 28 (**d**) relative to AAV injection. (**e**) Blood collected during the IPGTT on day 28 was assayed for circulating insulin. (**f**) Fasting insulin levels throughout the study were assessed by ELISA. Data shown as mean ± SEM (**a**–**f**) with individual traces (**b**–**f**). Pancreata collected six weeks post-AAV were immunostained for insulin and Cre (**g**). Insets show 4x enlargement of representative cells with bright insulin and Cre immunoreactivity, diminished insulin and Cre immunoreactivity, and cells in the core of the islet with neither insulin nor Cre immunoreactivity. (**h**) Pancreas was immunostained for insulin and IAPP. The intensity of insulin immunoreactivity in IAPP + cells was quantified, and individual cells for each animal are presented as violin plots with percent of cells below a designated cut-off (black line) shown as mean ± 95% confidence interval. IAPP immunoreactivity was used to calculate the β-cell area as a percent of total pancreas area and average islet β-cell area. (**i**) Pancreas was immunostained for insulin and CD3. Data analysed by repeated measures two-way ANOVA (**a**–**f**) or Mann-Whitney test (**h**). n = 4–5, scale bars are 100 μm, and insets are enlarged 4×. (**p < 0.01, ***p < 0.001).

To highlight the utility of the AAV8 Ins1-Cre as an alternative to Cre driver mouse lines for producing β-cell recombination, we delivered 1 × 10^12^ VGP IP to *Ins1*^−/−^;*Ins2*^*f*/f^ mice at 6–8 weeks of age. *Ins1*^−/−^;*Ins2*^f/f^ mice have deletions of the *Ins1* open reading frame and have LoxP sites flanking the *Ins2* gene thus making them inducible insulin knockouts^31^. This study is an ideal comparison to the transgenic mouse model approach carried out using *Ins1*^−/−^;*Ins2*^f/f^;*Pdx1*-Cre^ER^ mice^31^ wherein following delivery of tamoxifen there was ~99% recombination efficiency, mice developed hyperglycemia two weeks post-tamoxifen, and had reduced circulating insulin four weeks post-tamoxifen. Delivery of IP AAV8 Ins1-Cre did not impact body weight (Fig. 6a) and there was induction of variable hyperglycemia in 3–6 weeks (Fig. 6b). We performed an IPGTT at 10 (Fig. 6c) and 28 days (Fig. 6d) post-AAV and detected severe glucose intolerance by day 28. Analysis of blood collected during the day 28 IPGTT revealed no significant changes in fasting insulin levels, but a blunted glucose-stimulated insulin release (Fig. 6e). Despite hyperglycemia, we were unable to detect significant changes in fasting insulin levels throughout the study (Fig. 6f). Six weeks post-AAV, we collected pancreas in PFA and immunostained for Cre and found heterogenous Cre immunoreactivity that was discordant with insulin immunoreactivity (Fig. 6g). We identified many cells in the core of the islet lacking not only insulin immunoreactivity, but also Cre. In addition, we observed cells with bright nuclear Cre but enduring bright insulin immunoreactivity. We next immunostained for insulin and islet amyloid polypeptide (IAPP; Fig. 6h and Supplemental Fig. 4) and used IAPP as a marker of β-cells for subsequent analyses. Though a majority of β-cells retained some insulin immunoreactivity, there was an obviously fainter insulin immunoreactivity in many cells leading to a flattened distribution (Kurtosis: −0.28 to −1.60) and a trend towards a non-unimodal distribution (D = 0.00932, p = 0.2414)^32^ as shown in a violin plot of average intensity of insulin immunoreactivity in IAPP + cells. As AAV-injected animals had an altered distribution of insulin intensity, we defined a cut-off (black line) and there was a significant increase in percent IAPP + cells below the cut-off in AAV8 Ins1-Cre injected animals (35.3 ± 8.7%) compared to controls (6.3 ± 3.8%). There was no change to β-cell area (0.67 ± 0.13%) compared to controls (0.65 ± 0.07%) and no change to average β-cell area per islet (3224 ± 199 μm^2^) compared to controls (2634 ± 208 μm^2^). Next, given accumulation of DAPI + Ins- cells near islets (Supplemental Fig. 4) we immunostained for CD3 and detected heterogeneous immunoreactivity in AAV-treated animals (Fig. 6i).

## Discussion

Cre-LoxP technology has been used to great success to study the role of genes of interest in β-cell function, phenotype, and physiological outcomes^2^. To date, the field has relied on transgenic mouse lines to deliver Cre and inducible CreER proteins to cells of interest. Many Cre driver lines face caveats of off-target recombination^6–8^ and constitutive expression of Cre may have detrimental effects on β-cell development and function^4,5,13–15^. Additionally, inducible CreER lines are complicated by GH minigene inclusions within transgenes, leaky recombination before tamoxifen delivery, potential β-cell defects, and tamoxifen toxicity. As an alternative, we propose that the AAV could be used as a vector for delivery of Cre to β-cells.

AAV is non-pathogenic and simple IP delivery of AAV8 can infect tissues of interest, including the pancreas. We designed an AAV8 carrying Cre under control of a fragment of the rat *Ins1* promoter. Use of AAV8 Ins1-Cre enabled efficient β-cell recombination via the insulin promoter and given that we observed 0% recombination rates in PBS treated control animals (Fig. 3a), delivery of AAV8 Ins1-Cre was necessary for recombination events thus enabling temporal control over target gene recombination with zero-leak prior to delivery of AAV8 Ins-Cre. Additionally, use of the AAV allowed us to avoid Cre driver mouse lines that include a GH minigene, we did not need to deliver tamoxifen, and there were no deleterious effects of AAV8 Ins1-Cre on glucose-stimulated insulin secretion nor Ca^2+^ dynamics in response to glucose and KCl. In contrast, islets from Ins2-Cre transgenic mice have defects in Ca^2+^ dynamics in response to glucose and tolbutamide stimulation^25^. Though we did not assess the impact of AAV8 Ins1-Cre on rates of β-cell replication and apoptosis, given that use of AAV8 Ins1-Cre did not require tamoxifen nor any transgenes containing a GH minigene, and also avoided constitutive Cre expression starting in development, it is likely that use of AAV8 Ins1-Cre avoids these caveats of working with some Cre driver mice and verification would be worthwhile in follow-up studies. Despite observations that higher dosese of AAV8 Ins1-Cre may cause some defects in β-cell function based on observations of impaired glucose tolerance, excellent recombination efficiency using 1 × 10^12^ VGP was feasible and avoided obvious β-cell defects.

The packaging limitations of a double stranded AAV precluded the use of a long insulin promoter like the 8.5 kb used in the Ins1-Cre/ERT transgene^7^. Thus, we used a 410 bp *Ins1* promoter fragment because *Ins1* is more β-cell specific than *Ins2*, which is also expressed in neurons of the hypothalamus^33^ and in other tissues including the thymus^34^. We justified using this length of promoter because most of the major insulin promoter regulatory elements are contained in the −400 to +100 position of the transcriptional start site, including principle A boxes and GG boxes for Pdx1 interaction, cAMP responsive elements, the C1 element for MafA binding, and others^35^. When AAV8 Ins1-Cre was delivered IP there was off-target recombination in the hypothalamus, specifically the arcuate nucleus and dorsomedial nucleus of the hypothalamus, suggesting that the short *Ins1* promoter used here was inadequate to provide β-cell selective expression. Delivery of AAV via the pancreatic duct resulted in undetectable hypothalamic recombination partially because a lower dose (1 × 10^11^ VGP) could be used to achieve β-cell recombination; however, the same dose produced hypothalamic recombination when delivered IP, suggesting that the magnitude of systemic spreading is lower when delivered via the pancreatic duct. Surprisingly, both IP and ID delivery resulted in abundant recombination in the exocrine pancreas, other islet cells, and liver. Though the infection of AAV8 appears highest in the pancreas^22^ and certainly use of an insulin promoter would lead to the highest expression in β-cells, recombination is not a linear assessment of expression but rather a binary outcome. As a comparison, when the AAV carried GFP under control of a rat Ins1 promoter (Fig. 4g), any leaky GFP in hepatocytes or exocrine pancreas remained undetectable. There is heterogeneity in expression level of *Pdx1* in the mouse and human pancreas, including a small percentage (12/185) of acinar cells in human pancreas with *PDX1* transcript levels higher than the median of the β-cell population^36^. Thus, it is possible that acinar cells or non-β cells within islets with higher PDX1 were more likely to activate the rat Ins1 promoter fragment and undergo a recombination event, despite such cells not expressing detectable GFP following transduction with AAV8 Ins1-GFP. In related studies, others have observed activity of the insulin promoter in mouse liver using a 760 bp fragment of the rat insulin 1 promoter^37^. With less sensitive, non-binary, histological assays, there is even detectable proinsulin in many tissues including liver and adipose in models of diabetes^38^.

After delivery of AAV8 Ins1-Cre IP to mTmG mice, extensive review of immunostained sections of pancreas from all AAV-treated mice in the study did not show any peri-islet DAPI + INS+ collections of cells suggestive of insulitis (Fig. 3). This contrasted with studies on confetti mice treated with ID AAV8 Ins1-Cre that have obvious insulitis as well as IP AAV8 Ins1-GFP treated mice. Based on these results we hypothesized that induction of fluorescent protein expression in islets during adulthood can initiate insulitis whereas mice that have fluorescent protein expression throughout their lifespan (mT before recombination) are not prone. An alternative hypothesis is that both ID delivery as well as high dose IP AAV (5 × 10^12^ VGP AAV8 Ins1-GFP) could lead to insulitis as a result of a greater viral accumulation in the pancreas. Based on a lack of insulitis when mTmG mice were treated with AAV8 Ins1-Cre IP, we did not expect to detect insulitis in the follow-up study treating *Ins1*^−/−^;*Ins2*^f/f^ mice with AAV8 Ins1-Cre IP and thus did not include an AAV-treated *Ins1*^−/−^;*Ins2*^+/+^ group.

Though faced with the caveat of off-target recombination in the liver, exocrine pancreas, and hypothalamus, the AAV8 Ins1-Cre approach requires no tamoxifen, avoids use of Cre transgenes with a GH minigene, avoids developmental and functional defects of constitutive Cre expression, and precludes early recombination events prior to delivery. With an appreciation of both the benefits and caveats of using AAV8 Ins1-Cre, we demonstrate the utility of this tool by inducing hyperglycemia in *Ins1*^−/−^;*Ins2*^f/f^ mice. Likely off-target recombination was not concerning because there are no other tissues that could reasonably cause hyperglycemia following loss of the insulin gene. Recombination events that delete *Ins2* in tissues that do not express insulin (such as the liver) are inconsequential. Further, unlike the alternative approach using an inducible Pdx-CreER^31^ that would require extensive breeding and genotyping, we could generate these mice in a single round of breeding and proceed with our experiment in <3 months. As expected, *Ins1*^−/−^;*Ins2*^f/f^ mice injected with AAV8 Ins1-Cre developed hyperglycemia and had reduced glucose-stimulated insulin secretion. Similar to the previous study on the *Ins1*^−/−^;*Ins2*^f/f^;mTmG;PdxCreER model^31^, we saw only a modest reduction in insulin immunoreactivity despite induction of diabetes and reduced glucose-stimulated circulating insulin levels. Given that a > 90% pancreatectomy is required to induce diabetes in rodents^39^, the loss of functional β-cell mass must be greater than the observed ~1/3 reduction in insulin immunoreactive β-cells to cause hyperglycemia. Unexpectedly, we observed some insulitis in AAV-treated mice but there was no reduction in IAPP immunoreactive cells which reduces the likelihood of immune destruction of β-cells. Given a lack of a AAV-treated Ins1^−/−^;Ins2^+/+^ group we cannot conclusively determine if the AAV itself caused insulitis or if a downstream effect of a loss of Ins2 led to immune invasion. Our inability to separate out these two interpretations demonstrates that despite not detecting a significant impact of AAV8 Ins1-Cre on body weight, blood glucose, glucose tolerance, or glucose-stimulated insulin secretion of mTmG animals (Figs. 1–3), it is possible that AAV8 Ins1-Cre caused unanticipated cases of islet inflammation in Ins1^−/−^Ins2^f/f^ mice and perhaps AAV8 Ins1-Cre can produce confounding effects on other study outcomes depending on a multitude of factors including animal genotype. Therefore, in addition to PBS treated floxed controls, it is prudent to include AAV-treated unfloxed controls in the experimental design.

Given that AAV8 Ins1-Cre treated *Ins1*^−/−^*;Ins2*^*f/f*^ mice had a normal β-cell area paired with the surprising presence of cells with bright nuclear Cre and cytoplasmic insulin, it is likely that some cells had persisting insulin immunoreactivity despite successful recombination. Barring unlikely explanations like insulin endocytosis^40^, presumably a Cre+INS+ cell should exist only for a few days after recombination because any stored insulin prior to recombination should be secreted in response to hyperglycemia. Enduring insulin immunoreactivity can be partially attributed to abundant insulin storage and high stability of insulin mRNA^31^, but assuming recombination occurred near the time of AAV injection, this seems inadequate to explain persistent near normal insulin storage (by comparing brightness of immunoreactivity to control cells) six weeks after recombination during a period of hyperglycemia. For example, mouse islet insulin secretion is ~3% of content per hour at 10 mM glucose^41^ and though highly stable, insulin mRNA has a half life of only one day at low glucose and three days in settings of hyperglycemia^42^. Thus, an alternative hypothesis to explain the presence of cells with likely recombination events but enduring insulin immunoreactivity for weeks, is that following recombination, β-cells may dedifferentiate thus leading to proliferation^31^ and a loss of glucose-stimulated insulin secretion. Triple-omics assessment of islets isolated 6 days post-tamoxifen from *Ins1*^−/−^;*Ins2*^f/f^;mTmG;PdxCreER mice^31^ found down-regulation of secretory pathways like “Golgi vesicle transport”. At the same time-point, β-cells were GFP + and had insulin immunoreactivity comparable to controls, suggesting a loss of secretory pathways even prior to loss of all secretory granules. Additionally, others have reported enduring insulin immunoreactivity in models of β-cell dedifferentiation^43^. Overall, the AAV8 Ins1-Cre was a useful tool to generate this model of diabetes and repeat investigations using the AAV8 Ins1-Cre to clarify these observations are worthwhile.

Generation of a suitable cohort of Cre driver mice with a floxed target gene can take over a year, require dozens of cages, hundreds of genotyping experiments, and hundreds of hours of labor. AAV8 Ins1-Cre can be readily manufactured for a few thousand dollars, the cost to treat each mouse can be < $50 (1 × 10^11^ VGP ID), and suitable mice can be ordered directly from a rodent breeding facility or can be bred within the lab from a handful of breeders. Additionally, although current AAV manufacturing techniques that require adherent cell cultures and serum supplemental media are costly^44^, some recent advances permit serum free suspension cultures that could improve production efficiency and perhaps lower cost^45^. That being said, we acknowledge that ID delivery is technically challenging and an invasive procedure thus presenting unique challenges. Another alternative to using a Cre driver mouse line may be using adenoviral constructs, though we are unaware of any such attempts. Adenoviruses are inexpensive and relatively simple, but there has been limited efficiency at targeting the endocrine pancreas and the adenovirus causes a significant leukocyte response in the pancreas^46,47^, a major drawback when attempting to study a disease with an autoimmune component. Other technologies including lipid nanoparticles are being investigated for delivering genes to pancreatic tumors^48^ but this approach is new and there may be challenges achieving adequate efficiency of delivery.

We propose that the AAV8 Ins1-Cre approach described here could be used as a tool for expedited generation of inducible zero leak knockout of β-cell specific genes. This approach avoids inclusion of transgenes and delivery of tamoxifen but faces the caveat of off-target recombination in the hypothalamus, liver, and exocrine and endocrine pancreas. Though these caveats may be problematic for some study designs, when deleting genes whose predominant function is in β-cells, the approach is faster and comparably efficient to Cre driver line models. We highlight this point by inducing diabetes in *Ins1*^−/−^;*Ins2*^f/f^ mice. This approach has already been used successfully to study the role of a gene responsible for insulin exocytosis^49^ and we suggest that others consider using the AAV8 Ins1-Cre as a way to study the impact of gain or loss of function of β-cell specific genes on *in vivo* β-cell function.

## Methods

### Plasmid preparation

The AAV8 Ins1-Cre plasmid was generated from the dsAAV mouse Ins2-EGFP plasmid, kindly provided by Dr. Paul Robbins^22^. First, the 1.1 kb Ins2 promoter (with 5′ UTR) was excised with *BamHI* and *AgeI* and replaced with 410-bp of the rat Ins1 promoter with the 5′ UTR (*Ins1*; primers: *Ins1*-F: 5′-CACTGGATCCTGAGCTAAGAATCCAGCTATCAATAGAA ACT; *Ins1*-R: 5′-CACACAACCCCGTGTTGGAACAATGACCTGGAAGATAG). Next, the EGFP open reading frame (ORF) was excised with *AgeI* and *NotI* and the 4.9 kb vector fragment was gel-purified. The Cre ORF was generated by PCR amplification from an Addgene CMV-Cre vector (pBS185) with a Kozak sequence and *XmaI* site added upstream and a *NotI* site downstream. (primers: CreORF-F: 5′-CACACGCCCGGGGCCGCCACCATGTCCAATTTACTG ACCGTACACCAA; CreORF-R: 5′-CACACGCGGCCGCCTAATCGCCATCTTCCAGCAGGC). The PCR product was digested with *NotI* and *XmaI* and ligated into the 4.9 kb vector to yield the final dsAAV8 Ins1-Cre plasmid. The plasmid was sent to the Children’s Hospital of Philadelphia (CHOP) for manufacturing of high titre recombinant dsAAV by triple transfection system and purified by CsCl gradient^50^. AAVs were administered IP or via the pancreatic duct (intraductally; ID), as previously described^22,51,52^.

### Animal models

All procedures with animals were approved by the University of British Columbia Animal Care Committee and carried out in accordance with the Canadian Council on Animal Care guidelines. Eight-week-old C57BL/6 J mice were ordered from Jackson Laboratories (Bar Harbor, Maine). mTmG mice were generated in the lab of Dr. Liqun Luo^24^ (Stanford University, Stanford, CA) and ordered from Jackson Laboratories. Rosa26-LSL-Luciferase mice were generated in the lab of Dr. William Kaelin^53^ (Dana Farber Cancer Institute, Boston, MA) and ordered from Jackson Laboratories. Confetti mice were generated in the lab of Dr. Hans Clevers^30^ (Hubrecht Institute, Utrecht, Netherlands) and ordered from Jackson Laboratories. *Ins1*^−/−^ mice were generated in the lab of Dr. J Jami^54^ (Institut Cochin, Paris, France) and *Ins2*^f/f^ mice were generated in the lab of Dr. Massimo Trucco^34^ (University of Pittsburgh, Pittsburgh, PA). All mice employed in this study were housed with a 12 h light, 12 h dark cycle and had ad libitum access to chow diet (2918, Harlan Laboratories, Madison, WI). Adult (6–8 weeks old) male mice were used in all studies.

### Physiological tests and plasma assays

To assess body weight and blood glucose mice were fasted for four hours during the morning (9:00–13:00) prior to measurements using a One Touch Ultra glucometer (Life Scan Inc., Burnaby, Canada). For glucose tolerance tests, mice were fasted for four (IP) or six hours (oral) and then given a glucose load (2 g/kg of body weight). Blood was sampled from the saphenous vein and measured for glucose and insulin before injection (t = 0 min) and at different time points. Plasma insulin levels were determined by a Mouse Insulin Ultrasensitive enzyme-linked immunosorbent assay (ELISA; ALPCO Diagnostics, Salem, NH). Serum AST and ALT activities were measured using commercially available kits (Sigma-Aldrich, St. Louis, MO).

### *In vivo* luciferase imaging

Rosa26-LSL-Luciferase mice were injected with XenoLight RediJect D-Luciferin Ultra Solution (150 mg/kg) and imaged under isoflurane anesthesia using the IVIS® Imaging System Lumina Series. At study termination animals were injected with the luciferin solution then euthanized before rapid dissection and imaging.

### Islet isolation and cell culture

Hank’s balanced salt solution (HBSS) containing type XI collagenase (Sigma-Aldrich, St. Louis, MO) was employed to isolate pancreatic islets as previously described^55^. Briefly, collagenase solution (1000 U/mL) was injected into the common bile duct then the pancreas dissected out and digested at 37 °C for 11–14 min. Islets were then washed with iced-cold HBSS, filtered through a 70 μm cell strainer, handpicked, and cultured in RPMI 1640 (Sigma-Aldrich, St. Louis, MO). To infect islets *in vitro*, AAV was added to the culture medium at a specific multiplicity of infection (10^6^ VGP/cell or 10^7^ VGP/cell) and the medium was changed in 18–24 hours.

### Ca^2+^ imaging

Following 48 h culture at 37 °C and 5% CO_2_ on glass coverslips, pancreatic islets from infected wild-type C57BL/6 J mice were loaded with 5 μM Fura 2-AM for 30 min and imaged on a Zeiss Axiovert 200 M inverted microscope equipped with temperature-controlled stage and a FLUAR 20X objective (Carl Zeiss, Thornwood, NY) as previously described^25^. During the experiments, islets were continuously perifused with Ringer’s solution containing 144 mM NaCl, 5.5 mM KCl, 2 mM CaCl_2_, 1 mM MgCl_2_, 20 mM HEPES (adjusted to pH 7.35 by NaOH).

### Tissue harvest and immunohistochemical analysis

Tissues were harvested and fixed in paraformaldehyde (PFA) 4% overnight after transcardial perfusion with PBS for 2 min and ice-cold PFA 4% for 10 min. After fixation, tissues were washed in 70% ethanol and embedded in paraffin, for subsequent sectioning (5 µm thickness; Wax-It Histology Services, Vancouver, Canada). Brains were left in PBS containing 20% sucrose for 4 days and then frozen at −40 °C in 2-methylbutane prior to sectioning at 30 µm on a sliding microtome, in a one-in-six series through the rostrocaudal extent of the hypothalamus and immunostained by standard method^56^. For immunofluorescence of paraffin-embedded tissues, sections were deparaffinized in xylene and rehydrated in graded ethanol before heat-induced epitope retrieval in an EZ Retriever microwave oven (BioGenes, Fremont, CA; 95 °C for 15 mins in 10 mM citrate buffer with 0.05% Tween-20). We blocked slides in DAKO Protein Block, Serum Free (Dako, Burlington, Canada) and incubated overnight in primary antibody diluted in DAKO Antibody Diluent followed by incubation in secondary antibody for 1 hour at room temperature. Slides were mounted and counterstained with nuclear stain 4′,6-diamidino-2-phenylindole (DAPI) and VECTASHIELD^®^ Hard Set Mounting Medium with DAPI (Vector Laboratories, Burlingame, CA), and images were captured and analyzed with an ImageXpress^®^ Micro XLS System, controlled by MetaXpress^®^ High-Content Image Acquisition & Analysis Software (Molecular Devices Corporation, Sunnyvale, CA) with a scientific CMOS camera, a Nikon 20 × Plan Apo objective (NA = 0.75, 1-6300-0196; Nikon, Tokyo, Japan), and DAPI (DAPI-5060B), FITC (FITC-3540B), Cy3 (Cy3-4040B), Texas Red (TXRED-4040B), and Cy5 (Cy5-4040A) filter cubes. We performed quantification of histological images in the same software (MetaXpress^®^). We used an automated journal (multiwavelength cell scoring) that identifies all cells by DAPI fluorescence, then we defined parameters for cell diameter, total area, and intensity of IAPP or insulin immunoreactivity to identify IAPP + or insulin+ cells and IAPP + or insulin+ area. Finally we assessed the presence of fluorescent protein immunoreactivity in insulin+ or insulin- cells to assess recombination rate (Figs. 3a,d, and 4d), assessed the intensity of insulin immunoreactivity in IAPP + cells (Fig. 6h), and/or calculated the % IAPP + area relative to the total pancreas area using autofluorescence to detect the total pancreas area (Fig. 6h). All antibodies used are listed in Table S1.

### Statistical analysis

Data are expressed as mean ± SEM. Statistical analysis was performed using the Mann-Whitney test, one-way ANOVA, or repeated measures two-way ANOVA as indicated (GraphPad Prism, GraphPad Software Inc., La Jolla, CA, USA). p values <0.05 were considered significant. The dip test for unimodality was performed in R version 3.5.3^57^ using the statistical package diptest.

## Supplementary information

Supplementary information.
