# Supplementary material for: AAV8 Ins1-Cre can produce efficient β-cell recombination but requires consideration of off-target effects
Source: Sci Rep. 2020 Jun 29;10:10518. doi: 10.1038/s41598-020-67136-w (PMC7324556; doi:10.1038/s41598-020-67136-w)
Supplement: Supplementary file 1 — Supplementary information. [file 41598_2020_67136_MOESM1_ESM.docx]

**Supplemental information for:**

**AAV8 Ins1-Cre can produce efficient β-cell recombination but requires consideration of off-target effects**

Adam Ramzy^1^, Eva Tudurí^1,2,3^, Maria M. Glavas^1^, Robert K. Baker^1^, Majid Mojibian^1^, Jessica K Fox^1^, Shannon M O'Dwyer^1^, Derek Dai^4^, Xiaoke Hu^1^, Heather C. Denroche^1^, Nazde Edeer^1^, Sarah L Gray^5^, Cameron B Verchere^4,6^, James D. Johnson^1,4^, Timothy J. Kieffer^1,4^

^1^ Department of Cellular and Physiological Sciences, Life Sciences Institute, University of British Columbia, Vancouver, British Columbia, Canada.

^2^ Centro de Investigación Biomédica en Red de Diabetes y Enfermedades Metabólicas Asociadas (CIBERDEM), Spain.

^3^ Instituto de Investigación, Desarrollo e innovación en Biotecnología Sanitaria de Elche (IDiBE), Elche, Spain.

^4^ Department of Surgery, University of British Columbia, Vancouver, British Columbia, Canada.

^5^ Northern Medical Program, University of Northern British Columbia, Prince George, British Columbia, Canada.

^6^ Department of Pathology and Laboratory Medicine, BC Children’s Hospital Research Institute, Vancouver, British Columbia, Canada.

**Supplemental Figure 1. AAV Ins1-Cre does not elevate liver enzymes.** AST and ALT levels were measured in serum from PBS and AAV Ins1-Cre injected animals before (day 0) and on day 14 relative to virus delivery.

**Supplemental Figure 2. AAV Ins1-Cre can infect islets *in vitro.*** Islets from adult reporter mTmG mice were isolated and exposed to 10^6^ VGP and 10^7^ VGP of AAV Ins1-Cre per cell, or PBS, overnight. Endogenous EGFP fluorescence was tracked for the following four days.


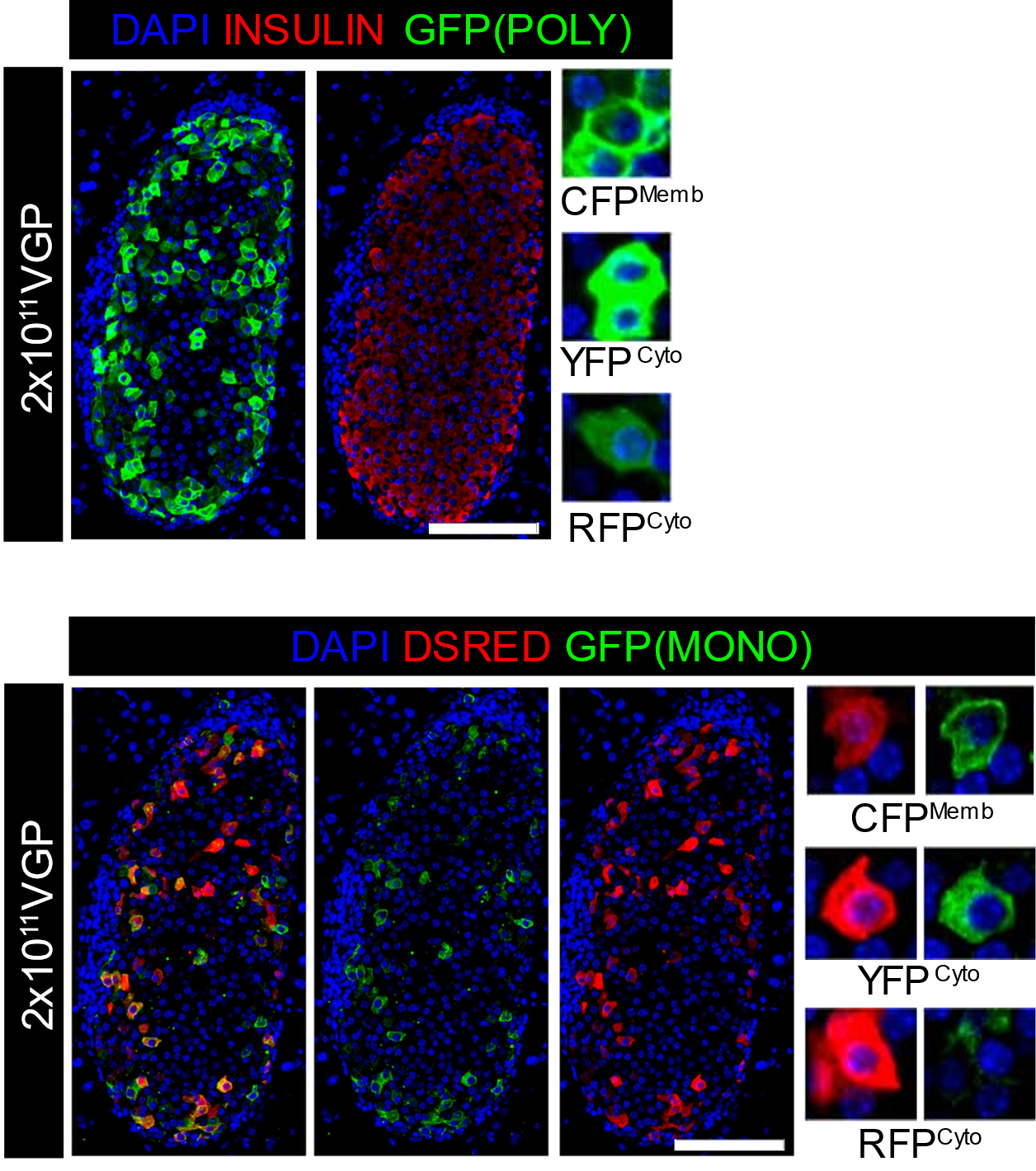


**Supplemental Figure 3. High sequence similarity allows detection of different confetti recombination events using a polyclonal α GFP antibody.** Pancreas collected from adult confetti mice treated with ID AAV Ins1-Cre was immunostained for fluorescent proteins to identify recombination events yielding nuclear GFP (not clearly observed), cytoplasmic RFP, cytoplasmic YFP, and membranous CFP. Representative images of n=3. Scale bar is 100 μm and insets are enlarged 4x.


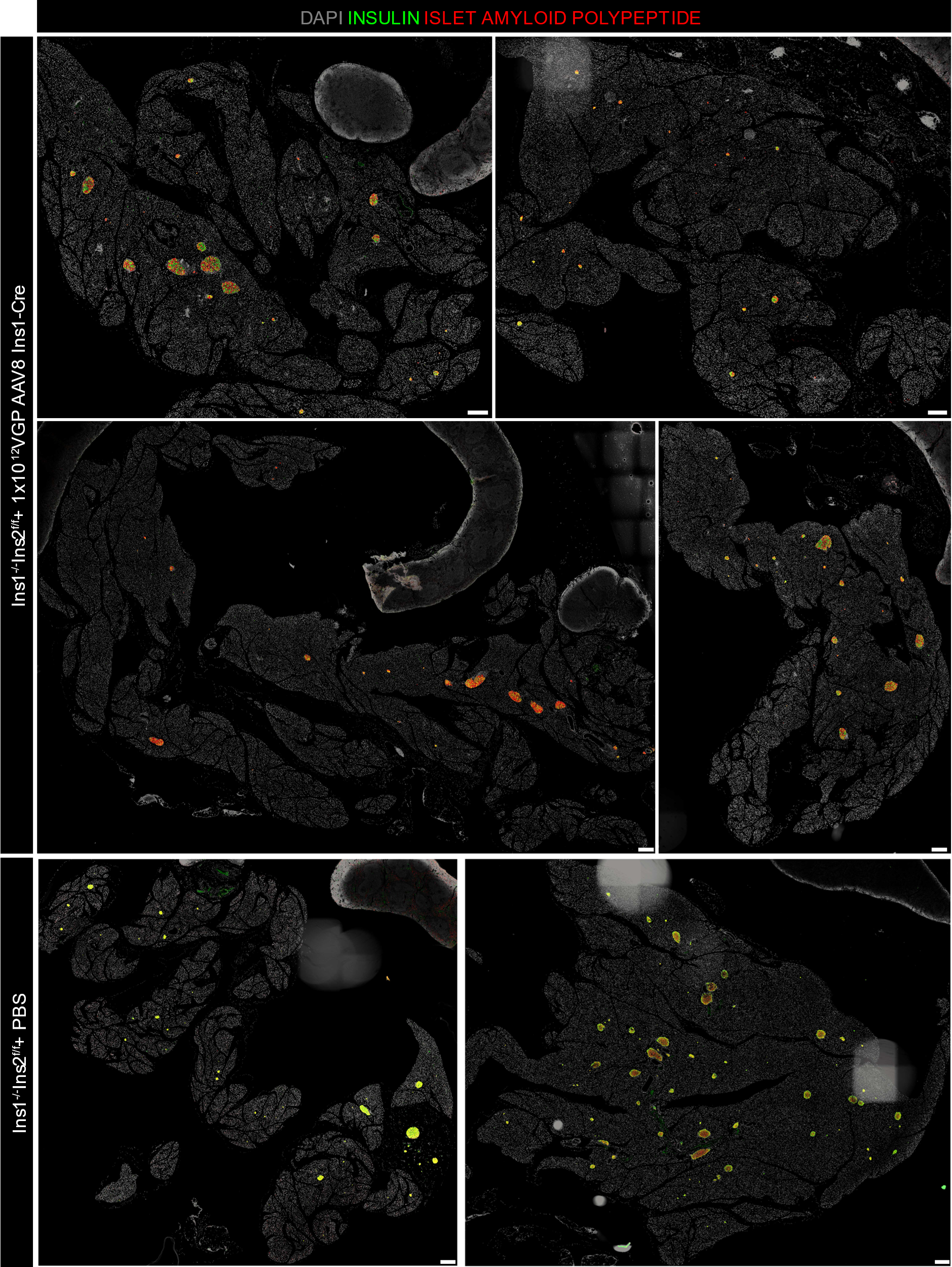


**Supplemental Figure 4. AAV8 Ins1-Cre treated *Ins1^-/-^;Ins2^f/f^* mice have a normal IAPP+ area but develop heterogenous insulitis.** Pancreas collected from *Ins1^-/-^;Ins2^f/f^* mice treated with either PBS or 10^12^ VGP AAV8 Ins1-Cre was immunostained for insulin and IAPP. Scale bars are 250 μm.

**Supplemental Table 1. List of primary antibodies used for Immunohistofluorescence.**

| **Peptide/protein target** | **Antigen Sequence** | **Name of Antibody** | **Manufacturer, catalog #, and/or name of individual providing the antibody** | | **Species raised in; monoclonal or polyclonal** | | **Dilution used** | **RRID** |
| --- | --- | --- | --- | --- | --- | --- | --- | --- |
| CD3 | C-terminus of human CD3 | Rabbit anti-CD3 | Anaspec | 29588 | Rabbit | Polyclonal | 1 to 50 | AB_2275572 |
| CD45 | Unknown | Rat anti-CD45 | BD Biosciences | 550539 | Rat | Monoclonal | 1 to 25 | AB_2174426 |
| dsRED | Unknown | Rabbit anti-dsRED | Clontech | 632496 | Rabbit | Polyclonal | 1 to 100 | AB_10013483 |
| Glucagon | unknown | Mouse anti-GCG | Sigma | G 2654 | Mouse | Monoclonal | 1 to 1000 | AB_259852 |
| Glucose transporter 2 | First extracellular loop of Glut2 | Rabbit anti-GLUT2 | Millipore | 07-1402 | Rabbity | Polyclonal | 1 to 500 | AB_1587076 |
| Green fluorescent protein | Full length *Aequorea* GFP | Mouse anti- GFP (mono) | Clontech | 632375 | Mouse | Monoclonal | 1 to 200 | AB_2756343 |
| Green fluorescent protein | GFP | GFP (poly) | Life Technologies | A11122 | Rabbit | Polyclonal | 1 to 500 | AB_221569 |
| Islet amyloid polypeptide | unknown | Rabbit anti-IAPP | AbCam | ab15125 | Rabbit | Polyclonal | 1 to 50 | AB_2295631 |
| Insulin | unknown | Rabbit anti-INS | Cell Signaling | C27C9 | Rabbit | Monoclonal | 1 to 200 | AB_2126503 |
| Insulin | Residues surrounding Val36 of human insulin | Mouse anti-INS | Cell Signaling | L6B10 | Mouse | Monoclonal | 1 to 250 | AB_10949314 |
| V-maf muscoloapo-neurotic fibrosarcoma oncogene homolog A | unknown | Rabbit anti-MAFA | Betalogics (Johnson & Johnson) | LP9872 | Rabbit | Polyclonal | 1 to 1000 | AB_2665528 |
| homeodomain transcription factor 6.1 | Human Nkx6.1 | Goat anti-NKX6.1 | R and D Systems | AF5857 | Goat | Polyclonal | 1 to 20 | AB_1857045 |
| Somatostatin | Human somatostatin | Mouse anti-SST | Β Cell Biology Consortium | AB1985 | Mouse | Polyclonal | 1 to 500 | AB_10014609 |
